# Long-Term Nitrogen Addition Leads to Loss of Species Richness Due to Litter Accumulation and Soil Acidification in a Temperate Steppe

**DOI:** 10.1371/journal.pone.0047369

**Published:** 2012-10-12

**Authors:** Ying Fang, Fen Xun, Wenming Bai, Wenhao Zhang, Linghao Li

**Affiliations:** 1 State Key Laboratory of Vegetation and Environmental Change, Institute of Botany, Chinese Academy of Sciences, Xiangshan, Beijing, China; 2 Graduate School of Chinese Academy of Sciences, Yuquanlu, Beijing, China; University of Tartu, Estonia

## Abstract

**Background:**

Although community structure and species richness are known to respond to nitrogen fertilization dramatically, little is known about the mechanisms underlying specific species replacement and richness loss. In an experiment in semiarid temperate steppe of China, manipulative N addition with five treatments was conducted to evaluate the effect of N addition on the community structure and species richness.

**Methodology/Principal Findings:**

Species richness and biomass of community in each plot were investigated in a randomly selected quadrat. Root element, available and total phosphorus (AP, TP) in rhizospheric soil, and soil moisture, pH, AP, TP and inorganic N in the soil were measured. The relationship between species richness and the measured factors was analyzed using bivariate correlations and stepwise multiple linear regressions. The two dominant species, a shrub *Artemisia frigida* and a grass *Stipa krylovii*, responded differently to N addition such that the former was gradually replaced by the latter. *S. krylovii* and *A*. *frigida* had highly-branched fibrous and un-branched tap root systems, respectively. *S. krylovii* had higher height than *A*. *frigida* in both control and N added plots. These differences may contribute to the observed species replacement. In addition, the analysis on root element and AP contents in rhizospheric soil suggests that different calcium acquisition strategies, and phosphorus and sodium responses of the two species may account for the replacement. Species richness was significantly reduced along the five N addition levels. Our results revealed a significant relationship between species richness and soil pH, litter amount, soil moisture, AP concentration and inorganic N concentration.

**Conclusions/Significance:**

Our results indicate that litter accumulation and soil acidification accounted for 52.3% and 43.3% of the variation in species richness, respectively. These findings would advance our knowledge on the changes in species richness in semiarid temperate steppe of northern China under N deposition scenario.

## Introduction

Nitrogen (N) is recognized as a primary factor limiting plant growth in many terrestrial ecosystems, and N fertilization has been widely used to stimulate plant growth and improve productivity [1], [2]. As a result, annual N input into terrestrial ecosystem has been increased from 34 Tg N yr^−1^(1860s) to 100 Tg N yr^−1^ (1990s) through the use of N fertilizer, N-fixation of legume plants, fuel combustion and other anthropogenic activities [3]. It is predicted that annual N input will reach to 200 Tg N yr^−1^ by the year of 2050 [3]. This increase in N input has great impacts on ecosystem N cycling [4], [5], ecosystem structures and other functions [6], [7], [8], [9].

The nutrient availability is an important factor to determine the species composition of vegetation [10], [11]. There are many reports demonstrating that N addition alters the community structure and composition in different terrestrial ecosystems. For instance, Bobbink et al. (1998) found that an increase of N availability in several vegetation types results in competitive exclusion of characteristic plant species by nitrophilic species [10]. Several dominant species including grasses and forbs usually dominate in a typical steppe in Inner Mongolia [12]. However, the gramineous *Stipa krylovii* becomes predominant after N fertilization for a few years [12]. Duprè et al (2010) analyzed data associated with long-term N in European grasslands, and they detected that N deposition significantly affects vascular plants, bryophytes and dicotyledon, leading to the loss of distinct dicotyledon [13]. However, few studies have addressed the reasons for the changes in individual species in response to N addition.

Species diversity is an important property for the community as it determines ecosystem productivity [14]. The knowledge about the mechanisms responsible for the loss of species diversity is crucial for the conservation of species [15]. Although many experimental studies found that species diversity is decreased after N addition [16]–[22], no mechanistic explanation has been given so far [21]. Moreover, the explanation for loss of distinct species after N addition remains unclear [22]. N addition has potential effects on ecosystems, including, acidification and eutrophication, and these factors may contribute to species diversity loss and changes in species composition [23], [24]. It has been suggested that light competition may account for the disappearance of some species because those species at the top of the canopy and/or with fast growth rate can preferentially use light resources [25]–[28]. There are also reports suggesting that loss of species diversity caused by N fertilization may result from disruption of nutrient absorption due to alteration of nutrient status in soil by soil acidification [13], [18], [29]. In addition, N fertilization can inhibit photosynthetic rate resulting from disturbance of nutrient balance in plants, leading to an increase in plant mortality [10], [30]. However, Lamb (2008) analyzed the effects of below-ground biomass, light availability, litter accumulation and other factors on grassland species richness using a structural equation modeling, and found that litter accumulation may account for the loss of species diversity [31]. Therefore, more experimental studies are required to clarify the primary mechanism underlying the N fertilization-induced loss of species richness.

To experimentally elucidate the mechanism by which loss of species richness occurs under N addition, a field experiment with five different N addition rates was conducted in a typical steppe in Inner Mongolian grassland. The following three questions were addressed. (1) How does long-term N addition affect community structure? (2) What are the mechanisms for species-specific responses? (3) Which is the primary factor to determine to the species loss after N fertilization?

## Methods

### Study Site

This study was conducted at the Duolun Restoration Ecology Station of the Institute of Botany, Chinese Academy of Sciences Experimental design in Duolun County (116°17′E, 42°02′N, 1324 m a.s.l.), Inner Mongolia, China. The area is located in the temperate climatic zone, and its mean annual temperature is 2.1°C with mean monthly temperature ranging from −17.5°C in January to 18.9°C in July [32]. Mean annual precipitation is 382.2 mm, and approx. 90% of the precipitation occurs from May to October. The soil in this area is classified as chestnut according to the Chinese classification and Haplic Calcisols based on the FAO classification [32]. Soil bulk density was 1.31 g cm^−3^ and pH was 6.84. Vegetation in this area is a typical steppe community and the dominating species are perennials, including *Stipa krylovii*, *Artemisia frigida*, *Potentilla acaulis*, *Cleistogenes squarrosa, Allium bidentatum, Leymus chinensiss, Carex korshinskyi, Salsola collina, Melilotoides ruthenica and Agropyron cristatum* [33].

### Experimental Design

The experimental area was fenced to exclude livestock grazing in July, 2003. A total of 64 plots of 15 m×10 m was established, and each of them was surrounded by a buffer strip with a width of 4 m. Eight treatments were included in our study, including one control treatment (no nutrient addition) and seven treatments of N enrichment at various levels. The experiment used a Latin square design to receive urea containing 46% N addition treatment on the middle growth time (July) of every year since 2003, by evenly spreading urea with hand. Eight N fertilization treatments, including 0 (N0), 1 (N1), 2 (N2), 4 (N4), 8 (N8), 16 (N16), 32 (N32) and 64 (N64) g N m^−2^_,_ were randomly assigned to 64 plots [12]. In our study, samples were collected from 40 plots with five treatments (N0, N2, N8, N16 and N32). The N treatments used in the present study include those N levels that are higher than the average farmland fertilization (10 g m^−2^) and the local N deposition rate (18 kg ha^−2^ y^−1^). The use of higher N levels allows us to evaluate long-term extreme N supply on steppe ecosystem.

### Investigation of Community Composition

Vegetation survey was conducted in the peak of biomass in mid-August of 2010. Species composition of community in each plot was investigated in a randomly selected quadrat (1 m × 1 m). Species richness was defined as the total species number per square meter [34], [17]. Aboveground biomass in every quadrat was clipped at the ground level, and both living and standing dead parts belonging to a same species were pooled together. Surface litter in the same quadrate was also collected. All plant samples including litter were oven-dried at 70°C for 48 h and then the biomass was determined separately. The height of each species within a quadrat was calculated as the average of species’ natural height from at least five random measurements [32].

### Measurement of Root Element, Available Phosphorus (AP) and Total Phosphorus (TP) in Rhizospheric Soil

Three individuals of *S. krylovii* or *A. frigida* were randomly selected in each plot in June, 2010, and were dug out with roots using a shovel. We chose *S. krylovii* and *A. frigida*, because they are two dominant species and most sensitive to N supply. A composite sample for each plot was obtained by combining three strains from every species. Plants were gently shaken by hand, and then rhizospheric soil was collected with a brush [35], [36]. Finally, all the roots from these three strains were excised with scissors. Roots were washed with de-ionized water and oven-dried at 70°C for 48 h. Dry roots were ground to pass through a 0.25-mm sieve, and then ground powder was solvented with Microwave Acceleration Reaction System (CEM Corporation, USA) after the digestion with H_2_SO_4_ and HNO_3_. Finally, contents of sodium (Na), calcium (Ca) and phosphorus (P), potassium (K) and sulfur (S) in the roots of *S. krylovii* and *A. frigida* were determined using an inductively coupled plasma emission spectrometer (Thermo Electron Corporation, USA). After the samples were digested with H_2_SO_4_-HClO_4_, total phosphorus contents in rhizospheric soil were determined by molybdenum-stibium colorimetry method [37] with a UV-visible spectrophotometer (UV-2550, SHIMADZU Corporation, China). To determine the available phosphorus contents, rhizospheric soil was ground with a mill and passed through a 0.25-mm sieve, and the filtered soil was digested with NaHCO_3_ [37].

### Measurement of Moisture, pH, TP, AP and Inorganic N in the Soil

In each plot, a soil core (3-cm diameter) of fresh soil from 0–10 cm soil layer was randomly sampled in June, July and August of 2010, respectively. Gravimetric method was used to determine the soil moisture. Soil samples were weighed before and after they were oven-dried at 105°C for 48 h. The mean value of three determinations was used to analyze the correlation between species richness and soil moisture. In mid-June, an additional soil core was sampled in the same way from each plot, and was used to measure the pH and concentrations of AP and TP and inorganic N. Some fresh soil in each soil core was extracted by 2 mol L^−1^ KCl solution (soil:KCl solution; 1∶10) [38] after passed through a 2-mm sieve, and then inorganic N concentrations were analyzed with Auto Analyzer 3 System (SEAL Analytical Gmbh, Germany). Here, inorganic N concentration was defined as the sum of NO_3_^–^N and NH_4_^+^-N concentrations. Some other air-dried soil from the same soil core was passed through a 2-mm sieve for determination of soil pH, and TP and AP concentrations. Soil pH was determined with Russell RL060P portable pH meter (Thermo Electron Corporation 166 Cummings Center, USA), and the water/soil ratio was 1∶2.5. Soil AP and TP concentrations were analyzed with the same method as for rhizospheric soil of *S. krylovii* and *A. frigida*.

### Statistical Analysis

One-way ANOVA (SPSS software package) was used to evaluate the effects of an 8-year-long N addition on soil parameters, plant biomass, plant height, species richness and root element contents. Pairs of mean values were compared with least significant difference (LSD). Bivariate correlations were used to determine the correlation of species richness with soil pH, litter amount, soil moisture, soil inorganic N concentrations and AP concentrations. Stepwise multiple linear regressions were further used to identify the most important factor affecting species richness after the 8-year-long N addition. All the statistical analyses were performed with SPSS software package (SPSS 16.0 for windows, SPSS Inc., Chicago, IL, USA). P value of less than 0.05 was considered as statistically significant.

## Results

### Effect of N Addition on Soil pH, Soil Moisture, Inorganic N Concentrations, and AP and TP Concentrations

Soil pH was significantly reduced by 9%, 12% and 18% in N8, N16 and N32 plots compared with the control, respectively (Table 1). Soil moisture in N16 and N32 plots was increased by 30% and 44% respectively compared with that in control (N0 plot). Soil inorganic N concentration in N8, N16 and N32 plots was 6, 7 and 10 times greater than that in N0 plot, respectively. In contrast, N addition had no effect on soil available phosphorus (AP) and total phosphorus (TP) concentrations (Table 1).

**Table 1:** Effects of long-term N addition on soil pH, soil moisture, and inorganic N concentration, soil available phosphorus (AP) and total phosphorus (TP) concentration in 0–10 cm soil layer.

Soil properties	N addition gradient (g N m^−2 ^y^−1^)				
	N0	N2	N8	N16	N32
pH	7.05±0.08a	6.87±0.04a	6.42±0.10b	6.18±0.08c	5.79±0.08d
Soil moisture(%)	5.97±0.35c	6.24±0.26c	7.13±0.34b	7.52±0.63b	8.61±0.51a
Inorganic N(mg Kg^−1^)	3.79±0.40b	8.65±1.30b	27.61±8.74a	30.90±5.31a	43.30±7.54a
AP (mg Kg^−1^)	3.15±2.11a	3.28±1.49a	3.65±2.74a	3.77±1.68a	3.73±3.16a
TP (mg Kg^−1^)	310.00±20.00a	301.25±20.22a	333.75±9.44a	305.00±13.23a	315.00±17.93a

All data were expressed as mean ± standard error (SE). Different letters within a column indicate the significant difference of mean values (*P*<0.05) in a row.

### Effect of N Addition on Biomass, Height, Root and Rhizospheric Soil Element Contents of *S. krylovii* and *A. frigida*

**Figure 1:**
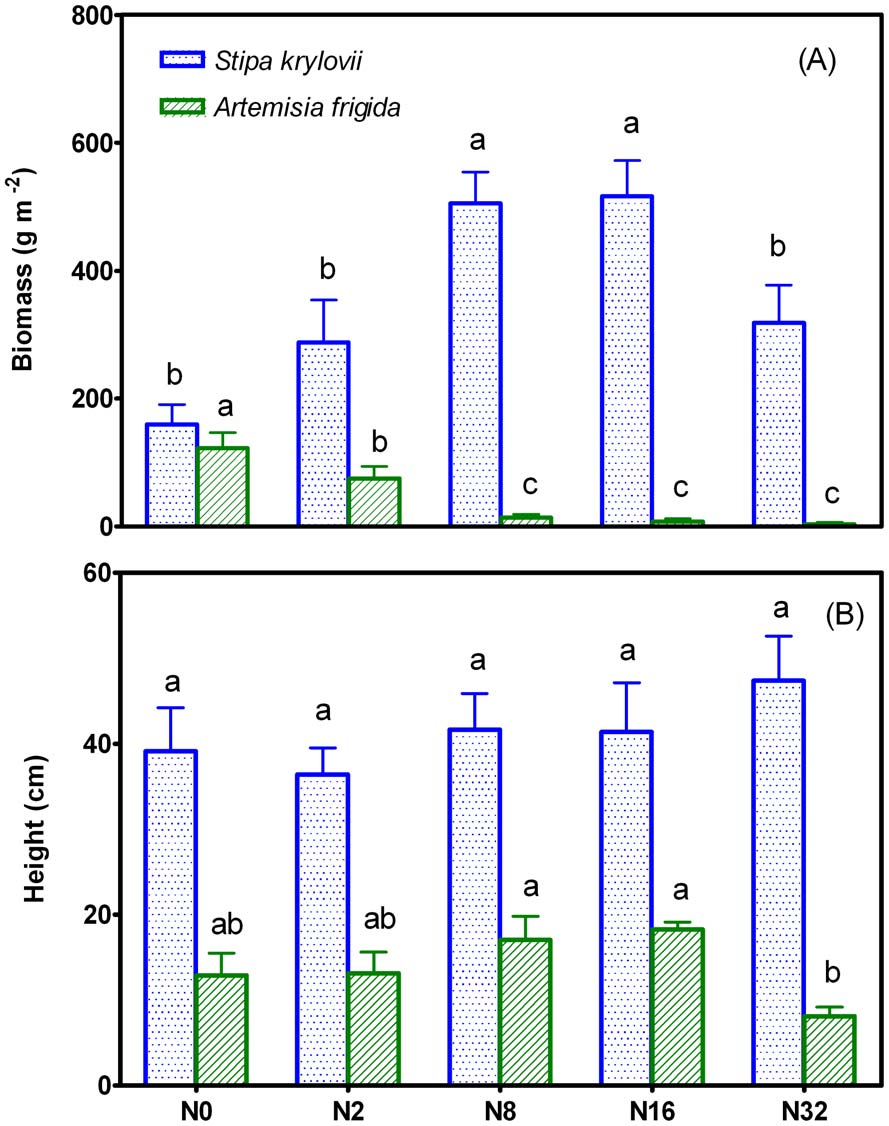
Biomass and height of *Stipa krylovii* and *Artemisia frigida* along 5 N addition treatments in 2010. All data were expressed as mean ± standard error (SE). Means that are significantly different are indicated with different letters (*P*<0.05).

There was a sigmoidal increase in aboveground biomass of *S. krylovii* with increases in N addition levels. For example, the biomass reached peak value in N8 and N16 plots, and declined to a level comparable to that in N0 plot when N level was further increased to N32 level. In contrast to *S. krylovii*, a linear decrease in biomass of *A. frigida* with increases in N addition levels was observed (Fig. 1A). For example, the biomass in N2, N8, N16 and N32 plots was decreased by 39%, 89%, 94% and 97%, respectively (Fig. 1A). In contrast to biomass, N addition had no impact on plant height of *S. krylovii* (Fig. 1B). There were no significant differences in plant height of *A. frigida* among N0, N2, N8 and N16 plots, but plant height in N32 plot was significantly lower than that in other 4 N added plots (Fig. 1B).

Root Ca content of *S. krylovii* in N8, N16 and N32 plots was reduced by 13%, 18% and 20% compared with that in N0 (Table 2). Root Ca content of *A. frigida* in N2 and N32 plots was increased by 24% and decreased by 19% compared with that in N0 plot, respectively (Table 2). Moreover, root P content of *S. krylovii* in N2 plot was increased by 12% compared with that in N32 plot. However, no significant differences were observed in root P content of *A. frigida* among the five N addition levels (Table 2). For *S. krylovii*, root Na content in N8 and N16 plots was decreased by 22% and 24% compared with that in N32 plot, respectively. For *A. frigida*, root Na content in N2 and N8 plots was increased by 104% and 138% compared with that in N0 plot, respectively (Table 2). In addition, No significant differences in root K and S contents among the five N addition levels in both *S. krylovii* and *A. frigida* were observed (unpublished data).

**Table 2:** Root element contents and rhizospheric soil available phosphorus (RAP) contents of *Stipa krylovii* and *Artemisia frigida* along 5 N addition treatments.

Plant species	Element contents (%)	N addition gradient (g N m^−2 ^y^−1^)				
		N0	N2	N8	N16	N32
*Stipa krylovii*	Ca	0.826±0.016a	0.799±0.030 a	0.721±0.025b	0.675±0.018b	0.657±0.029b
	P	0.055±0.001ab	0.057±0.002 a	0.053±0.001ab	0.054±0.001ab	0.051±0.002b
	Na	0.011±0.001ab	0.011±0.0004ab	0.011±0.001b	0.010±0.001b	0.013±0.001a
	RAP	0.004±0.0002c	0.004±0.0001c	0.004±0.0003bc	0.005±0.0002b	0.005±0.0003a
*Artemisia frigida*	Ca	0.633±0.038b	0.784±0.034a	0.653±0.028b	0.605±0.034b	0.515±0.015c
	P	0.068±0.003a	0.066±0.004a	0.072±0.003a	0.067±0.004a	0.064±0.003a
	Na	0.006±0.001b	0.012±0.003a	0.014±0.001a	0.010±0.001ab	0.010±0.002ab
	RAP	0.005±0.0001b	0.005±0.0002b	0.006±0.0002a	0.006±0.001a	0.006±0.0001a

All data were expressed as mean ± standard error (SE). Different letters within a column indicate the significant difference of mean values (*P*<0.05) in a given plant species.

We also found that rhizospheric AP contents of *S. krylovii* and *A. frigida* were responsive to N fertilization (Table 2). Rhizospheric AP contents of *S. krylovii* in N16 and N32 plots were increased by 18% and 37% compared with that in N0 plot, respectively. A similar change in rhizospheric AP contents of *A. frigida* was also found in response to N addition. For instance, rhizospheric AP contents of *A. frigida* N8, N16 and N32 plots were increased by 30%, 30% and 19% (Table 2) compared with those in N0 plot, respectively.

### Aboveground Biomass and Species Richness of Community

**Figure 2:**
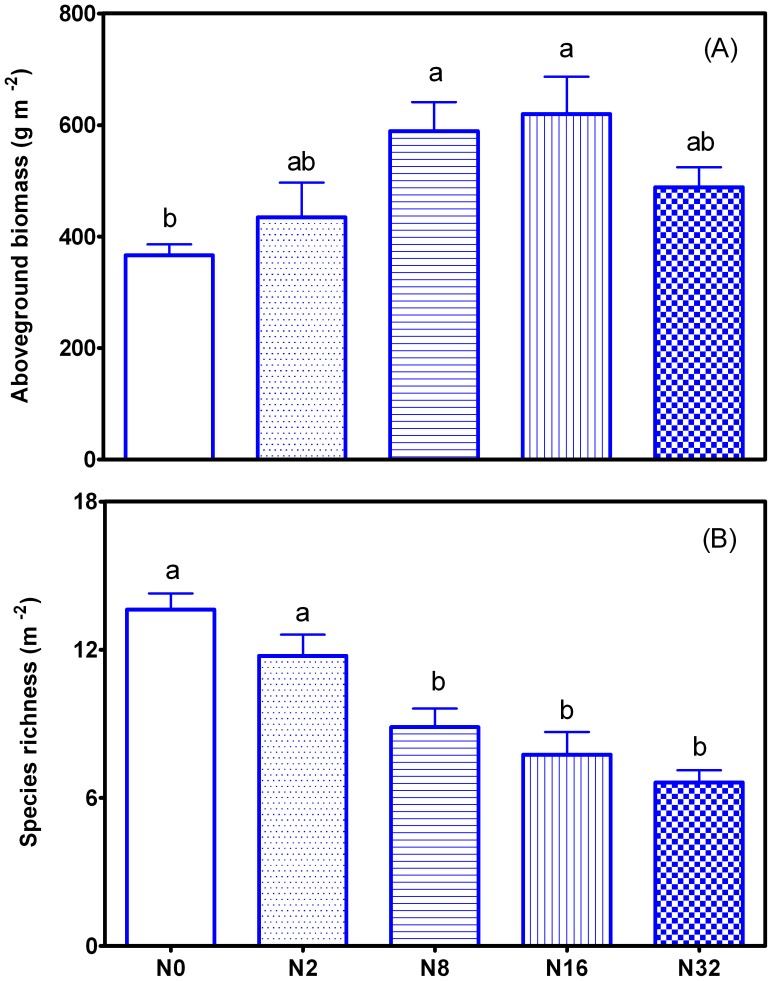
Aboveground biomass and species richness of community along 5 N addition treatments in 2010 (A, MEAN±SE, n = 8). All data were expressed as mean ± standard error (SE). Means that are significantly different are indicated with different letters (*P*<0.05).

The aboveground biomass of community in N8 and N16 plots was increased by 61.04% and 69.32% compared with that in N0 plots, respectively. Further increase in N to N32 level led to a reduction in the aboveground biomass of community such that the biomass in N32 plot was not significantly different from that in N0 and N2 plots (Fig. 2A). A linear decease in species richness with increases in N addition levels was observed (Fig. 2B).

### Correlations between Species Richness and Soil pH, Litter Amount, Soil Moisture, Soil AP Concentration, and Inorganic N Concentration

**Figure 3:**
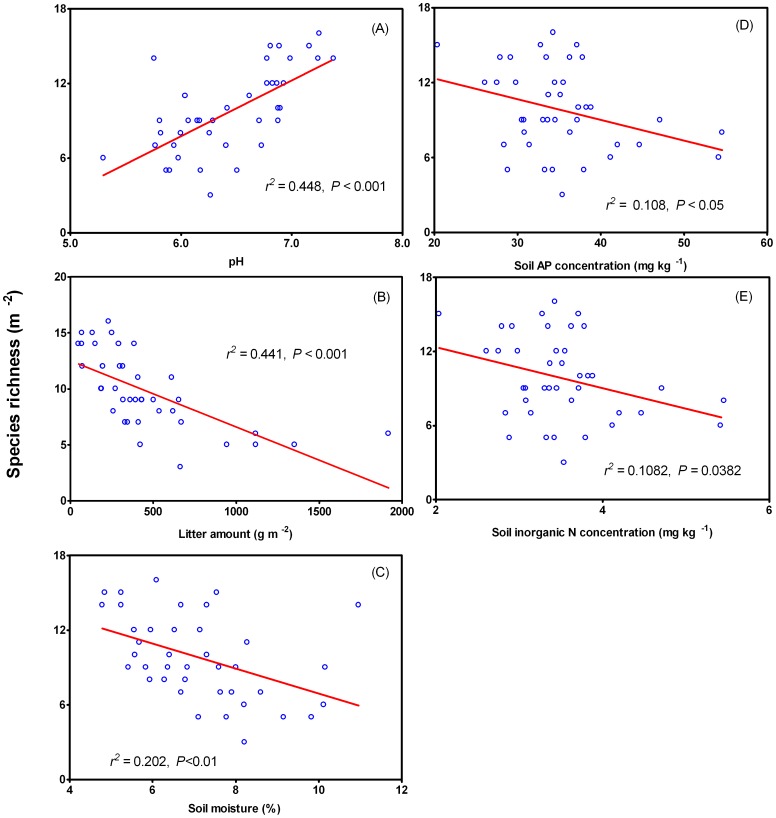
Species richness and correlation with soil pH (A), litter amount (B), soil moisture (C), soil AP concentration (D) and inorganic N concentration (E).

Species richness was positively correlated with soil pH (Fig. 3A), while a negative correlation between species richness and litter amount (Fig. 3B), soil moisture (Fig. 3C), AP concentration (Fig. 3D) and inorganic N concentration (Fig. 3E) was observed. Stepwise multiple regression analyses revealed that litter amount and soil pH accounted for 52.3% (*r^2^* = 0.523, *P* = 0.0001) and 43.3% (*r^2^* = 0.433, *P* = 0.0001) of variations in species richness after long-term N addition in semi-arid steppe, respectively.

## Discussion

### Community Structure Changes after Long-term N Addition

In the present study, we found that long-term N addition had contrasting effect on biomass of the two dominant species, *S. krylovii* and *A. frigida* such that biomass of *S. krylovii* and *A. frigida* was enhanced and suppressed by N addition, respectively (Fig. 1). As a shrub, *A. frigida* was gradually replaced by *S. krylovii,* a grass species in response to N addition. The mechanism underlying this change remains to be dissected. The difference in the fine root morphology between the two species may contribute to the community change. *S. krylovii* has a highly-branched fibrous root system that is mainly distributed in the soil surface, whereas *A. frigida* has an unbranched tap root system that can reach deep soil [12]. The fibrous root system may have advantage to absorb more surface soil nutrient than tap root system. Huang et al. (2008) showed that N use efficiency of *S. krylovii* is increased after N addition [12]. In addition, as light has an important effect on community, we thus investigated the effect of N addition on plant height of the two species. Our results revealed that *S. krylovii* was higher than *A. frigida* in both control and N addition plots (*P* < 0.05). No effect of N addition on *S. krylovii* height was found, while an inhibitory effect on *A. frigida* height was observed in N32 plot exclusively (Fig. 1). It has been widely accepted that species with high height preferentially would capture more light resource than species with short height, and that the short height species would tend to be excluded from light competition [25], [26], [27], [28]. A similar argument may also be used to explain the replacement of *A. frigida* by *S. krylovii* in plots with high levels of N addition. We found that N addition had significant impact on root Ca content in both *S. krylovii* and *A. frigida* such that Ca content was decreased with the increased amount of N (Table 2). Our result is consistent with previous reports that Ca^2+^ loss is stimulated by N deposition [39], [40]. This observation is partly because redundant N loses from soil occurs in the forms of NO_3_^−^ and Ca^2+^, and other basic cations eluviate to imbalance electric charges [39], [41]. Another reason may be that N enrichment results in an increase of NH_4_^+^ concentration, thus leading to reduction in the absorption of Ca^2+^ and other basic cations since many plants absorb NH_4_^+^ first [30]. The alteration of nutrient balance may have negative effects on plant growth [42], [43]. The root Ca contents in *S. krylovii* were higher than those in *A. frigida* in plots supplemented with N addition (Table 2). These results suggest that the two species may have distinct strategies for Ca acquisition, and that *S. krylovii* may be more effective to acquire Ca than *A. frigida*. Finally, the N addition induced a greater loss of Ca in *A. frigida* than in *S. krylovii* (Table 2). This may lead to damages to *A. frigida*, thus accounting for the replacement of *A. frigida* by *S. krylovii*. Another important observation in the present study is that long-term N addition resulted in acidification and increases in activities of rhizospheric AP (Table 2). More specifically, we found that N addition-induced change in root P content in *S. krylovii* was more evident than that in *A. frigida*, suggesting that *A. frigida* may be more sensitive to change soil P availability. This feature may facilitate growth of *S. krylovii* under conditions of N addition. The N addition-induced increase in Na content in *A. frigida* was greater than that in *S. krylovii* (Table 2). This result indicates that the greater accumulation of Na may have a toxic effect on *A. frigida*, thus disrupting a number of physiological processes associated with growth and development [44]–[46].

### Species Richness Loss after Long-term N Addition

We found that species richness was significantly reduced after an 8-year-long N fertilization (Fig. 1). This result is consistent with those reported in the literature [8], [11], [17], [18], [20], [28]. Moreover, our result showed that N addition significantly induced soil acidification, and that species richness was positively correlated with soil pH. It is conceivable that some plant species would not adapt to the acidic soil due to alteration of nutrient balance in soil, thus leading to disruption of nutrient acquisition by plants [10], [47]. We observed that litter accumulation was increased with the increased amount of N, and that species richness was negatively correlated with the litter accumulation. This result may be accounted for by that a deep litter layer would decrease light intensities at ground surface in the community, thereby suppressing seed germination, inhibiting seedling establishment and increasing the mortality of other small plants [48], [49]. This effect may finally result in the loss of species richness. We also found that soil moisture was increased with the increase in N addition rate, and that species richness was negatively correlated with soil moisture. Litter accumulation may improve soil moisture by retaining more water or reducing water loss [50]. In the same area, it has been reported that plant productivity is limited by water, therefore improvement of soil moisture would contribute to plant growth [33]. However, these positive effects cannot offset the negative effects caused by litter accumulation. As a consequence, long-term N addition would lead to the loss of species richness.

We found that species richness was negatively correlated with soil AP concentrations, although the AP concentration was not significantly affected by long-term N addition. Previous studies reported that P plays an important role in plant growth under N sufficient conditions. It has been shown that N addition at a low rate enhances C and P accumulation, thus improving P use efficiency and absorption efficiency, while a high N addition rate has no effect or even inhibits P accumulation and P use efficiency [51], [52]. Our results revealed that species richness was negatively correlated with concentration of soil inorganic N. An increase in inorganic N concentration imposes a positive effect on soil nutrients, resulting in an increase in plant productivity [53], [54], [55]. However, N addition increases the competition between community species [26], [56]. Huang et al. (2008) found that N-resorption efficiency (NRE) of *S. krylovii* is most sensitive to changes in soil N regime. This may explain its success over other species at a wide range of soil N availability. For example, height of *S. krylovii* was higher than that of *A. frigida* in the absence and presence of N addition. The stimulatory effect of N addition on *S. krylovii* height would reduce light and other resources available to other small plants. Finally, *S. krylovii* may be of more advantage to compete for resources than other species, thus leading to a decline in species richness. A similar result has been reported in the literature [26], [56].

Our results from the stepwise multiple linear regression analyses indicate that litter accumulation and soil acidification were the two primary factors for the loss of species richness in a typical temperate steppe after long-tem N addition (Fig. 3). Lamb (2008) confirmed that litter accumulation is the primary mechanism responsible for species richness in grassland by structural equation modeling. However, the modeling budget is different from the experimental results. Our findings in the present study provide some experimental evidence indicating that litter accumulation and soil acidification may underlie the loss of species richness by long-term N addition in grassland ecosystems.

### Conclusion

Atmospheric N deposition is continually increased due to frequent anthropogenic activities, fuel combustion and usage of fertilizers. Therefore, the effects of N deposition on terrestrial ecosystems have attracted much more attention in recent years. In this study, we found that long-term N addition caused the changes in community structure in temperate steppe, Inner Mongolia, China, and that shrub *A. frigida* was largely replaced by the dominant graminoid, *S. krylovii*. Species richness was notably decreased with the increase in amount of N addition. We further showed that the loss of species richness after long-term N fertilization may be accounted for by litter accumulation and soil acidification.
